# Parasites of domestic and wild animals in South Africa. LI. Ticks infesting leopard tortoises *Stigmochelys pardalis*, hingeback tortoises *Kinixys zombensis* and angulate tortoises *Chersina angulata*

**DOI:** 10.4102/ojvr.v84i1.1303

**Published:** 2017-02-28

**Authors:** Ivan G. Horak, Ashley Pearcy, Kyle J. Lloyd

**Affiliations:** 1 Department of Veterinary Tropical Diseases, University of Pretoria, South Africa; 2 Animal, Plant and Environmental Sciences, University of Witwatersrand, South Africa; 3 Department of Zoology and Entomology, Rhodes University, South Africa

## Abstract

The objective of the study was to record the tick species collected from three species of tortoise, each in a different province of South Africa. Ticks were collected from leopard tortoises, *Stigmochyles pardalis,* in the southern region of the Kruger National Park, Mpumalanga province; from hingeback tortoises, *Kinixys zombensis,* in the Enseleni Nature Reserve, KwaZulu-Natal province and from angulate tortoises, *Chersina angulata,* in the West Coast National Park, Western Cape province. Of the 63 leopard tortoises examined, 58 were infested with *Amblyomma marmoreum* and 49 with *Amblyomma hebraeum,* and all stages of development of both species were recovered. *Amblyomma nuttalli* was collected from 25 hingeback tortoises, and all stages of development were present. All 24 angulate tortoises examined were infested with *Amblyomma sylvaticum,* and large numbers of larvae, nymphs and adults were collected. Three snake species and a sand lizard were also infested with *A. sylvaticum*. The adults of *A. marmoreum, A. nuttalli* and *A. sylvaticum* were identified as specific parasites of the family Testudinidae, whereas all stages of development of *A. hebraeum* were classified as generalists.

## Introduction

The history of ticks on tortoises in South Africa dates back to the 18th century. The first tick with a strictly South African distribution to be described was collected from a tortoise by Sparrman during his travels in the Cape (Theiler 1943). This new species was described as *Acarus sylvaticus* (now known as *Ablyomma sylvaticum*) by De Geer in 1778. Ticks on tortoises were again reported by Arbousset and Daumas in the 1846 account of their exploratory tour of the Cape of Good Hope (Walker & Schulz 1984). The majority of ticks that have been documented on tortoises in South Africa belong to four species of *Amblyomma* and all are ornate. Theiler (1943) published illustrations and a description of *Amblyomma sylvaticum,* and Theiler and Salisbury (1959) illustrated and described ticks of ‘the *Amblyomma marmoreum* group’, including *Amblyomma nuttalli* and *A. marmoreum*, whereas Walker and Olwage (1987) have produced colour illustrations of *Amblyomma hebraeum* and *A. marmoreum*. The sexes of the four *Amblyomma* species are dimorphic and the males are the most brightly coloured.

Within South Africa, *A. marmoreum* is the most widespread of the four species and is present in all nine provinces, although only patchily in some (Horak et al. 2006a). It is most prevalent in the Fynbos, Albany Thicket and Savanna biomes and the eastern region of the Nama Karoo biome. Relatively few collections have been made in the large Grassland biome of South Africa. Although *A. hebraeum* is not regarded as a tick of tortoises, it is regularly found on leopard tortoises within its own distribution range from the Albany Thicket biome in the south of the Eastern Cape province through the Savanna biome of the east and northern regions of the country to the eastern region of North West province (Spickett 2013). The third species, *A. nuttalli,* is widespread in Africa, but has a distribution virtually restricted to the Indian Ocean Coastal Belt biome in north-eastern KwaZulu-Natal (Horak et al. 2006b). The core distribution of the fourth species, *A. sylvaticum,* lies within the Fynbos biome of the Western and Northern Cape provinces (Horak et al. 2006b).

Extensive host records for *A. marmoreum* have been provided by Horak et al. (2006a). All stages of development infest leopard tortoises, *Stigmochyles pardalis*, as well as various other tortoise species, whereas the larvae, and to a lesser extent nymphs, infest carnivores, equids, bovids, leporids and ground-frequenting birds. The usual hosts for all stages of development of *A. hebraeum* are large herbivores, whereas the larvae and nymphs also infest small herbivores, carnivores, hares and ground-frequenting birds (Horak et al. 1987). Walker and Schulz (1984) collected *A. hebraeum* nymphs from 13 of 29 leopard tortoises and from 4 of 7 angulate tortoises, *Chersina angulata* examined in the southern region of the Albany Thicket biome, whereas Dower, Petney and Horak (1988) collected *A. hebraeum* nymphs and a few adults from 14 leopard tortoises in the same biome. Walker and Schulz (1984) also collected the adults of *A. hebraeum* from a leopard tortoise in the Savanna biome in north-eastern KwaZulu-Natal. Horak et al. (2006b) recorded nine males of *A. nuttalli* on five of seven hingeback tortoises, *Kinixys zombensis,* examined in the Indian Ocean Coastal Belt biome. They also recorded fairly large numbers of *A. sylvaticum* in all stages of development on 124 of 138 angulate tortoises and a few on other tortoise species examined in the Fynbos biome (Horak et al. 2006b).

Norval (1975) studied the life cycle of *A. marmoreum* in the laboratory, feeding all stages of development on tortoises. Including the time the ticks spent off-host while moulting and laying eggs, and using the maximum period of feeding for each life stage, the life cycle took 242 days to complete. However, when the larvae and nymphs were fed on sheep, the life cycle could be completed in 193 days. *Amblyomma marmoreum* larvae detached from naturally infested tortoises between 8 and 104 days after capture, nymphs between 4 and 47 days, males between 1 and 111 days and females between 1 and 73 days (Dower et al. 1988). Norval (1974) also studied the life cycle of *A. hebraeum* in the laboratory, feeding the larvae on rabbits, the nymphs on sheep and the adults on calves. On these hosts, the life cycle was completed between 114 and 165 days, with the larvae feeding for 4–15 days before detaching, the nymphs for 6–9 days and the females for 6–12 days. A single female tick laid 18 765 eggs (Norval 1974). However, on naturally infested tortoises, *A. hebraeum* nymphs spent 1–125 days (mean 40 days) before detaching, males 14–92 days (mean 56 days) and females 15–77 days (mean 49 days) (Dower et al. 1988). Aeschlimann (1967) fed all stages of development of *A. nuttalli* on tortoises, and the life cycle took between 130 and 196 days to complete. According to him, Santos Dias in Mozambique counted 22 891 eggs laid by a single female tick. No studies have been conducted on the life cycle of *A. sylvaticum*.

Fielden and Rechav (1994) recorded most *A. marmoreum* larvae (49.6%) on the head and neck of leopard tortoises, followed by the anterior legs and the anterior and posterior armpits. Most nymphs attached to the head and neck (53.6%), followed by the anterior legs. Most males attached to the posterior armpit (35.3%), followed by the posterior legs and around the base of the tail and on the tail, and most females attached around the base of the tail and on the tail (47.8%), followed by the posterior legs and the posterior armpit. Pearcy and Beyer (2013) report that the larvae and nymphs of *A. sylvaticum* attach to the soft tissue around the base of the neck and the sockets of the front and hind limbs of angulate tortoises. Most males attach on the outer shell between the scutes and the remainder on the body of the tortoise, whereas approximately equal numbers of females attach to the shell and to the body. There are no attachment data for *A. nuttalli*.

The aim of this study was to record collections of ticks from tortoises subsequent to the publications of Horak et al. (2006a, 2006b). Here we report on collections of *A. marmoreum* and *A. hebraeum* from leopard tortoises, of *A. nuttalli* from hingeback tortoises and of *A. sylvaticum* from angulate tortoises and explore the possibility of host specificity amongst the four ticks. The likelihood of transmission of diseases to domestic livestock is also discussed.

## Methods

Ticks were collected by A.P. (University of Witwatersrand) from a single Speke’s hingeback tortoise, *Kinixys spekii,* and 63 leopard tortoises between Skukuza, Lower Sabie and Pretoriuskop in the southern Kruger National Park between September 2011 and February 2013. Ticks were also collected by A.P. from 24 angulate tortoises at Duinepos in the West Coast National Park over a 5-day period between 27 and 31 October 2012 (Pearcy & Beyer 2013). The sites of attachment of all stages of development on the angulate tortoises were recorded. Ticks were collected by K.J.L. (Rhodes University) from 25 hingeback tortoises in the Enseleni Nature Reserve in north-eastern KwaZulu-Natal between 03 December 2012 and 28 January 2013. The ticks collected from each animal were placed in separate vials containing 70% ethyl alcohol with a pencil-written label providing collection data. The ticks were identified and counted by I.G.H. (University of Pretoria) using a stereoscopic microscope. The tortoise species sampled, and the species and numbers of ticks recovered have been summarised in Table 1.

**TABLE 1:** Ixodid ticks collected from three tortoise species in three regions of South Africa.

Tick and tortoise species	Number infested	Number of ticks collected			

		Larvae	Nymphs	Males	Females
**Savanna biome (Mpumalanga)**					
Leopard tortoises (*n* = 63)	-	-	-	-	-
*Amblyomma marmoreum*	58	381	446	225	51
*Amblyomma hebraeum*	49	19	245	13	1
**Indian Ocean Coastal Belt biome (KZN)**					
Hingeback tortoises (*n* = 25)	-	-	-	-	-
*Amblyomma nuttalli*	25	1	31	92	19
**Savanna biome (Mpumalanga)**					
Speke’s hingeback tortoise (*n* = 1)	-	-	-	-	-
*Amblyomma nuttalli*	1	0	0	1	0
**Fynbos biome (Western Cape Province)**					
Angulate tortoises (*n* = 24)	-	-	-	-	-
*Amblyomma sylvaticum*	24	3200	377	289	63
*Amblyomma sylvaticum* (other hosts)	-	-	-	-	-
Centipede eater (*Aparallactus capensis*)	1	20	0	0	0
Mole snake (*Pseudaspis cana*)	1	26	5	3	2
Common egg eater (*Dasypeltis scabra*)	1	8	0	0	0
Spotted sand lizard (*Pedioplanis lineoocellata*)	1	2	0	0	0

KZN, KwaZulu-Natal province.

Cumming (1998) considered that an Afrotropical ixodid tick had to infest at least 50 host species to be considered a generalist. For a tick to be considered a specialist at least 10 collections had to be made and at least 90% of all collections had to come from a single taxon. Using these criteria, Cumming (1998) classified *A. hebraeum* as a generalist. We, however, decided to decrease the number of host species infested to 20 for a tick to be considered as a generalist, instead of the 50 species Cumming suggested for the whole of Africa. We only consider ticks as specialists if at least 20 collections had been made and at least 90% of all collections were made from a single taxon. Furthermore, animals of at least four other species unrelated to the taxon of specificity were examined at the same locality or localities.

## Ethical considerations

The South African National Parks Research Committee approved the projects for the capture of tortoises and removal of ectoparasites. Fieldwork in the Enseleni Nature Reserve was conducted under a permit issued by Ezemvelo KZN Wildlife (SCRP151) and Rhodes University Ethical Standards Committee (ZOOL-16-2012).

## Results

Fifty-eight out of 63 leopard tortoises examined in the southern Kruger National Park were infested with *A. marmoreum* and 49 with *A. hebraeum* (Table 1). One tortoise was not infested with either species, whereas 49 were infested with both, 13 with *A. marmoreum* only and four with *A. hebraeum* only. Fifty-six tortoises were infested with the adults of *A. marmoreum* and eight with the adults of *A. hebraeum. Amblyomma marmoreum* males outnumbered females by 4.4 to 1. All 25 of the hingeback tortoises examined in the Enseleni Nature Reserve were infested with *A. nuttalli*. Only one larva was collected, and male ticks outnumbered females by 4.8 to 1 (Table 1). A single *A. nuttalli* male was collected from a Speke’s hingeback tortoise in the southern Kruger National Park. The 24 angulate tortoises examined in the West Coast National Park were all infested with *A. sylvaticum,* and substantial numbers of larvae, nymphs and male ticks were collected. Males outnumbered females by 4.6 to 1 (Table 1). Three species of snake and a sand lizard were also infested with *A. sylvaticum*.

According to our adaptation of Cumming’s approach, and making use of all the data available to us from Horak and his co-worker’s past surveys, the adults of *A. marmoreum, A. nuttalli* and *A. sylvaticum* are specialists on the family Testudinidae, whereas the immature stages of *A. marmoreum* and all stages of development of *A. hebraeum* are generalists. More than 95% of collections of adult *A. marmoreum* were made from Testudinidae, whereas several species within six other families, unrelated to Testudinidae, were examined for ticks in the same localities. Besides Testudinidae, the immature stages of *A. marmoreum* were collected from more than 60 other species unrelated to Testudinidae. More than 95% of collections of adult *A. nuttalli* were made from Testudinidae, whereas nyalas (*Tragelaphus angasii*), bushbuck (*Tragelaphus scriptus*), red duikers (*Cephalophus natalensis*) and bushpigs (*Potamochoerus larvatus*) were examined within the same region (Horak, Boomker & Flamand 1991, 1995). More than 95% of collections of adult *A. sylvaticum* were made from Testudinidae, whereas eland (*Tragelaphus oryx*), gemsbok (*Oryx gazella*), bontebok (*Damaliscus pygargus pygargus*), springbok (*Antidorcas marsupialis*) and hyraxes (*Procavia capensis*) were examined in the same national park as the angulate tortoises from which *A. sylvaticum* was collected (Golezardy & Horak 2007). The adults of *A. hebraeum* were collected from more than 25 species of mammals and the immature stages from more than 40 species of mammals as well as from five species of birds.

## Discussion

### Amblyomma marmoreum

Horak et al. (2006b) recorded 80 collections of *A. marmoreum* from 82 leopard tortoises, and we now add a further 58 collections from 63 leopard tortoises. The comparatively small number of larvae and nymphs recovered is not unusual. Even with careful scrutiny, the numbers of larvae and nymphs on leopard tortoises were underestimated by Rechav and Fielden (1995). These stages, and especially the larvae, infest leopard tortoises, other tortoise species, several reptile species and a variety of other hosts (Horak et al. 2006a). Very few adult ticks have been recovered from any hosts other than tortoises, and using a modified version of Cumming’s (1998) approach, we have classified the adults as specialists on Testudinidae, whereas the immature stages are generalists. Amongst the Testudinidae, more collections of *A. marmoreum* adults have been made from leopard tortoises than from any other species of tortoise (Horak et al. 2006b; Table 1).

Rechav and Fielden (1995) reported that the male to female ratio of *A. marmoreum* adults differed depending on the season during which leopard tortoises were examined in the National Zoological Gardens, Pretoria. They found that although there was an increase in numbers of both sexes between September and October (spring), the male to female ratio was constant at approximately 2:1, whereas in April (autumn) females were virtually absent and the ratio was approximately 20:1. The ratio of males to females on leopard tortoises examined in the Albany Thicket biome in the Addo Elephant National Park during September and October was 1.8:1 (Walker & Schulz 1984), 2.8:1 on tortoises captured between February and May in the same biome close to Grahamstown (Dower et al. 1988), 4.1:1 on tortoises examined during February in the Bontebok National Park in the Fynbos biome in the Western Cape province (Horak & Boomker 1998) and 4.4:1 on the tortoises examined in this study. These differences could be because of seasonal fluctuations in the abundance of male and female ticks, differences in the length of time that males or female ticks remained attached to their tortoise hosts, or because of the diligence with which ticks were collected.

Bezuidenhout (1988) demonstrated experimentally that leopard tortoises could be infected with *Ehrlichia ruminatium*, the causative organism of heartwater in domestic and wild ruminants, and that *A. marmoreum* could acquire infection from an infected tortoise. Later Peter, Burridge and Mahan (2000) showed that *A. marmoreum* larvae could acquire infection from goats with clinical heartwater and that after moulting the nymphs could transmit infection to susceptible sheep. The larvae of *A. marmoreum* are generalists and naturally feed on a large variety of animals (Horak et al. 2006a). It is thus possible that they could acquire infection while feeding on an infected ruminant, and the ensuing nymphs transmit infection to a susceptible animal. It would, however, appear that few nymphs infest domestic or wild ruminants (Horak et al. 1987). Consequently, the role that *A. marmoreum* might play in the epidemiology of heartwater in the field has yet to be determined.

### Amblyomma hebraeum

The adults of *A. hebraeum* are generalists, and besides tortoises, they infest equids, suids and large bovids (Horak et al. 1987). The immature stages are also generalists and infest the same hosts as the adults, but in addition feed on leporids and ground-frequenting birds (Horak et al. 1987). However, few larvae or adult ticks are found on tortoises (Horak et al. 2006b; Table 1). Cumming (1998) also classified *A. hebraeum* as a generalist but without discriminating between the immature stages and the adults. The larvae, whose mouthparts are long and relatively slender, possibly do not infest tortoises because their skins are too impenetrable (the mouthparts of *A. marmoreum* larvae are shorter and more robust than those of *A. hebraeum*). Adult ticks possibly do not infest tortoises because they prefer warm-blooded hosts. Too few adult ticks were collected to determine a meaningful sex ratio.

*Amblyomma hebraeum* is the most effective vector of *E. ruminantium* in South Africa. Infection can be acquired by larvae feeding on an infected host and transmitted by the ensuing nymphs, or acquired by nymphs and transmitted by adult ticks. If indeed tortoises are naturally infected with *E. ruminantium*, infection could be acquired from them by nymphs of *A. hebraeum* and transmitted to susceptible livestock by the ensuing adults. Whether this occurs in nature has yet to be proved.

### Amblyomma nuttalli

Despite extensive surveys conducted on a variety of host species in many of the national and provincial nature reserves, including north-eastern KwaZulu-Natal, Horak and his co-workers had never collected *A. nuttalli* (Horak et al. 1991, 1995), whose adults we regard as specialists on Testudinidae. *Amblyomma nuttalli* is widespread in the Afrotropical region (Burridge 2001), whereas its core distribution in South Africa is north-eastern KwaZulu-Natal, where Theiler (1962) records 10 collections. Aeschlimann (1967) reports that the distribution of *A. nuttalli* is associated with forests in Côte d’Ivoire. With the exception of the Knysna and Alexandria Forests biomes in the Western and Eastern Cape provinces, the Indian Ocean Coastal Belt biome in north-eastern KwaZulu-Natal is possibly the closest to a forest habitat in South Africa. Extralimittally adult and immature ticks have been recovered from tortoises, varanid lizards, a python and from puffadders and gaboon adders, whereas the immature stages have been collected from small forest dwelling antelopes, reptiles and ground-frequenting birds (Aeschlimann 1967; Burridge 2001; Theiler 1962).

### Amblyomma sylvaticum

Of the four *Amblyomma* species, only *A. sylvaticum* has a strictly South African distribution, and even in South Africa, its distribution is confined to the coastal and adjacent inland regions of the Northern, Western and western region of the Eastern Cape provinces. Although angulate tortoises appear to be the hosts of choice for all stages of development, *A. sylvaticum* is known to infest other tortoise species, and the immature stages infest lizards and snakes (Horak et al. 2006b; Walker 1991; Table 1). Amongst the Testudinidae, more collections of the adults of *A. sylvaticum* have been made from angulate tortoises than from any other species of tortoise (Horak et al. 2006b). Walker (1991) reports a collection of nymphs and adult ticks from a mole snake, *Pseudaspis cana* and here we record a collection of all stages of development from this snake. Mole snakes are large and sturdy and live underground in abandoned animal burrows and are prone to long fasts (Branch 1998). It is perhaps this behaviour that renders them more prone to infestation with adult ticks than other snakes.

### General

With the exception of *A. hebraeum*, of which too few adult ticks were collected, the male to female ratios presently recorded for the three other *Amblyomma* spp. were remarkably similar. The ratio for *A. marmoreum* was 4.4:1, for *A. nuttalli* 4.8:1 and for *A. sylvaticum* 4.6:1. The similarity might be fortuitous but generally reflects the fact that male ticks remain attached for longer than females (Dower et al. 1988; Norval 1975), leading to a preponderance of males. The gender ratios also affirm that the collections were thorough, in that female ticks are generally larger than males, and hence, they should theoretically be easier to see and collect. However, the high ratio of males to females reflects careful collection of the smaller males.

## Conclusion

Four of the nine *Amblyomma* species that are present in South Africa infest tortoises. Leopard tortoises are one of the preferred hosts of *A. marmoreum* adults, hingeback tortoises are one the preferred hosts of *A. nuttalli* adults, and angulate tortoises are one the preferred hosts of *A. sylvaticum* adults. The adults of these three species are specialist parasites of the family Testudinidae. The fourth species, *A. hebraeum*, which usually infests warm-blooded animals, frequently infests leopard tortoises within its own distribution range, and its adults and immature stages are generalists.
